# Androgen production, uptake, and conversion (APUC) genes define prostate cancer patients with distinct clinical outcomes

**DOI:** 10.1172/jci.insight.183158

**Published:** 2024-10-22

**Authors:** Hannah E. Bergom, Ella Boytim, Sean McSweeney, Negar Sadeghipour, Andrew Elliott, Rachel Passow, Eamon Toye, Xiuxiu Li, Pornlada Likasitwatanakul, Daniel M. Geynisman, Scott M. Dehm, Susan Halabi, Nima Sharifi, Emmanuel S. Antonarakis, Charles J. Ryan, Justin Hwang

**Affiliations:** 1 Masonic Cancer Center, and; 2 Department of Medicine, University of Minnesota-Twin Cities, Minneapolis, Minnesota, USA.; 3 Glickman Urological and Kidney Institute, Cleveland Clinic, Cleveland, Ohio, USA.; 4 Department of Clinical and Translational Research, Caris Life Sciences, Phoenix, Arizona, USA.; 5 Desai Sethi Urology Institute, Sylvester Comprehensive Cancer Center, University of Miami, Florida, USA.; 6 Department of Medicine, Siriraj Hospital, Mahidol University, Bangkok, Thailand.; 7 Fox Chase Cancer Center, Philadelphia, Pennsylvania, USA.; 8 Department of Biostatistics and Bioinformatics, Duke Cancer Institute, Durham, North Carolina, USA.

**Keywords:** Oncology, Bioinformatics, Molecular genetics, Prostate cancer

## Abstract

**BACKGROUND:**

Prostate cancer (PC) is driven by aberrant signaling of the androgen receptor (AR) or its ligands, and androgen deprivation therapies (ADTs) are a cornerstone of treatment. ADT responsiveness may be associated with germline changes in genes that regulate androgen production, uptake, and conversion (APUC).

**METHODS:**

We analyzed whole-exome sequencing (WES) and whole-transcriptome sequencing (WTS) data from prostate tissues (SU2C/PCF, TCGA, GETx). We also interrogated the Caris Precision Oncology Alliance (POA) DNA (592-gene/whole exome) and RNA (whole transcriptome) next-generation sequencing databases. Algorithm for Linking Activity Networks (ALAN) was used to quantify all pairwise gene-to-gene associations. Real-world overall survival was determined from insurance claims data using Kaplan-Meier estimates.

**RESULTS:**

Six APUC genes (*HSD3B1*, *HSD3B2*, *CYP3A43*, *CYP11A1*, *CYP11B1*, *CYP17A1*) exhibited coalescent gene behavior in a cohort of metastatic tumors (*n* = 208). In the Caris POA dataset, the 6 APUC genes (APUC-6) exhibited robust clustering in primary prostate (*n* = 4,490) and metastatic (*n* = 2,593) biopsies. Surprisingly, tumors with elevated APUC-6 expression had statically lower expression of *AR*, AR-V7, and AR signaling scores, suggesting ligand-driven disease biology. APUC-6 genes instead associated with the expression of alternative steroid hormone receptors, *ESR1/2* and *PGR*. We used RNA expression of *AR* or APUC-6 genes to define 2 subgroups of tumors with differential association with hallmark pathways and cell surface targets.

**CONCLUSIONS:**

The APUC-6-high/*AR*-low tumors represented a subgroup of patients with good clinical outcomes, in contrast with the *AR*-high or neuroendocrine PCs. Altogether, measuring the aggregate expression of APUC-6 genes in current genomic tests identifies PCs that are ligand (rather than AR) driven and require distinct therapeutic strategies.

**FUNDING:**

NCI/NIH 1R37CA288972-01, NCI Cancer Center Support P30 CA077598, DOD W81XWH-22-2-0025, R01 CA249279.

## Abstract 2

Androgen production, uptake, and conversion (APUC) genes define prostate cancer patients with ligand-driven disease and distinct clinical outcomes.

## Introduction

In advanced prostate cancer (PC), persistent activity of the androgen receptor (AR) is a key driver of tumor progression, patient survival, and metastases (1, 2). Numerous aberrancies leading to the retained signaling of AR have been described, including non-hormonal drivers, promiscuity of other steroid hormone receptors (SHRs), amplification or mutation in the receptor itself, and, to a lesser extent, variation in the levels of ligands that lead to AR activation. In most cases, death from PC is marked by retained and persistent AR activity. Therefore, further exploration of novel inputs to its activity is needed.

Understanding the relationship between measurable androgens and patient outcomes is nascent. Previous studies have found positive associations between higher serum levels of androgens and patient outcomes in settings in which androgen-lowering drugs are subsequently deployed (2–4). Opposed to serum levels, intratumoral androgen production is a driver of tumor progression (5, 6), and our understanding of its relationship to tumor status and patient outcomes is incomplete. Androgens measurable in the serum typically reflect an adrenal source, whereas the technical feasibility of quantifying tumor-produced androgens is currently not possible. Alternatively, the availability of intratumoral sequencing techniques now enables the study of such production.

Androgens may enter tumor cells directly or be produced within the tumor through conversion of precursors to androgen molecules. This occurs through an enzymatic process involving a suite of more than 20 genes that govern androgen production, uptake, and conversion (APUC). Given the essential need for these steroid molecules or ligands in driving tumor growth, variation in such genes may significantly impact outcomes in PC, either singularly or in aggregate. One critical APUC gene is *CYP17A*1, which encodes cytochrome P450 17A1. This enzyme converts pregnenolone and progesterone to 17-OH pregnenolone and 17-OH progesterone and 17-OH-pregnenolone to DHEA primarily in the adrenal glands. Such steroid intermediates are critical precursors of testosterone and dihydrotestosterone (DHT). Testosterone and DHT are extremely potent activators of AR, which led to the pharmacologic development of abiraterone (2). Insights into androgens of adrenal (7) and/or intratumoral origin (6) drove the clinical development of the androgen synthesis inhibitor abiraterone acetate. This therapy is a standard-of-care treatment for patients with advanced PCs across many clinical states because it directly targets CYP17A1, preventing the generation of testosterone and DHT (2, 8–10). Beyond abiraterone, opevesostat, which inhibits CYP11A1, is the only other drug specifically designed to ablate the activity of an APUC gene that is available for patients with PC (11).

Germline genetic studies further support the examination of the clinical impact of APUC genes in PC. The APUC protein 3β-hydroxysteroid dehydrogenase-1 (encoded by *HSD3B1*) is primarily expressed in the peripheral, non-endocrine tissues of the body and catalyzes the conversion of DHEA into androstenedione and other downstream androgens. The protein product of *HSD3B1* regulates the production of non-testicular testosterone or DHT (12), which are high-affinity ligands that activate AR. One variant of *HSD3B1*, in which sustained androgen synthesis is achieved through enzyme stabilization, is associated with resistance to AR-targeted therapies and subsequently unfavorable clinical outcomes (12–15). Beyond *HSD3B1*, variants of other APUC genes have been implicated in poor patient outcomes (16, 17). This includes the genes encoding the organic ion transport proteins, *SLCO2B1* (18, 19), *CYP19A1* (20), *CYP3A4* (21, 22), and *SRD5A2* (23), which results in the conversion of testosterone to DHT, creating the most potent AR agonist. Altogether, previous studies have demonstrated that germline genetic dysregulations in APUC genes can lead to poor patient outcomes, which suggests that intratumoral changes such as RNA levels measured by RNA-seq could also drive AR activation and therapy resistance.

The impact of intratumoral changes in either DNA or RNA of APUC genes is not well characterized. With 21 potential APUC genes, a challenge for defining their relationship to PC is that many intratumoral changes are detected in advanced prostate tumors and they are often not mutually exclusive. To address this and similar challenges, we previously developed the Algorithm for Linking Activity Networks (ALAN) (24), a bioinformatics tool that interprets gene networks within transcriptomic patient data by quantifying the relationship between all genes of interest. Given the complexity of APUC genes, and their many possible interactions, we sought to apply this unbiased approach to determine the intratumoral relationship between APUC genes within large PC patient datasets. In this study, we utilize a hypothesis-driven analysis of the associations of 21 genes known as enzymatic regulators of APUC (16) to determine their relationship PCs.

## Results

### Six APUC genes exhibit concordant behavior in metastatic PC.

**Figure 1:**
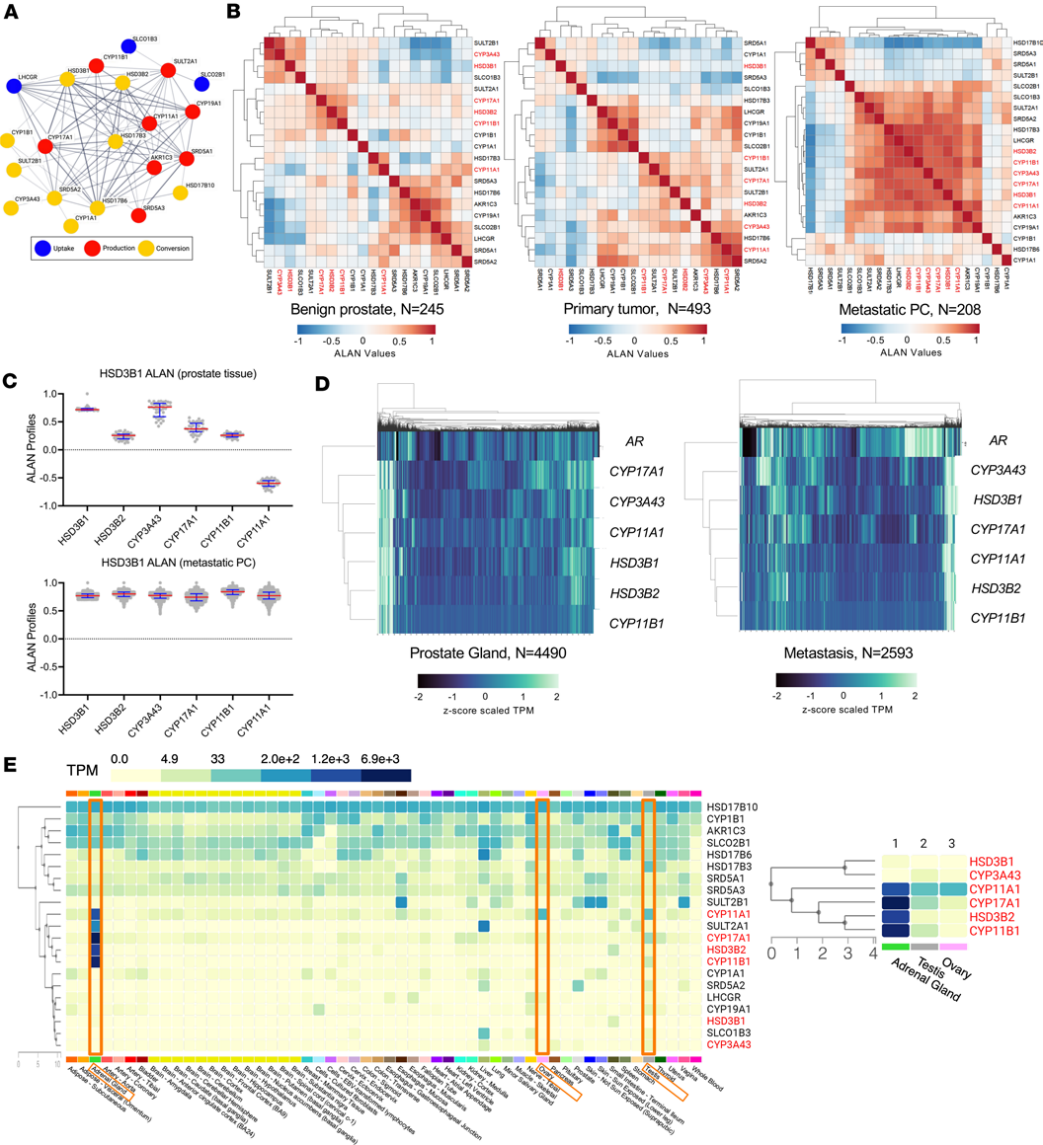
Interaction of APUC genes in prostate tissue. (**A**) STRING analysis was performed to indicate the degree of connection. Based on the output for molecular interactions, we then labeled uptake (blue), production (red), and conversion (yellow) genes. (**B**) The relationship between APUC genes was examined using ALAN outputs, values between –1 (blue) and 1 (red), based on WTS data from benign prostate tissue, PC, and metastatic PC tumors. Unsupervised hierarchical clustering was performed on ALAN outputs within each dataset. Six APUC genes are highlighted (red font). (**C**) The ALAN profiles for 6 APUC genes of interest are examined with greater detail in prostate tissue and metastatic PC. (**D**) Using WTS data from the Caris dataset, we conducted unsupervised hierarchical clustering of prostate and metastatic PC samples based on *z* score–scaled TPM data. (**E**) The median expression (TPM) of all APUC genes was examined in the GTEx database across all available tissue sites. Six APUC genes are highlighted (red font).

We first sought to utilize unbiased approaches to study the interplay between the 21 APUC genes. We used STRING (25) to conduct an iterative search in public databases in order to assess the strength of pairwise gene interactions (Figure 1A). While this confirmed that the family of APUC genes is largely associated with one another, we were unable to deconvolute which APUC gene interactions are relevant for the pathogenesis of PC. We next applied ALAN to quantify all pairwise gene-to-gene interactions based on a continuous measurement (i.e., RNA expression), which we previously utilized to model the changes in AR interactions in benign prostate tissue, primary PC, and metastatic PC (24). We performed ALAN on the transcriptomes from samples annotated as benign prostate tissue (Genotype-Tissue Expression [GTEx] project, *n* = 245) (26), primary PC (The Cancer Genome Atlas [TCGA], *n* = 493), and a cohort of metastatic PCs (Stand Up to Cancer/Prostate Cancer Foundation [SU2C/PCF], *n* = 208) (27). Unsupervised clustering of ALAN outputs indicated that 6 out of 21 APUC genes, *HSD3B1*, *HSD3B2*, *CYP11A1*, *CYP11B1*, *CYP17A1*, and *CYP3A43*, exhibited increasing association with respect to disease progression from benign to primary PC, but only exhibited robust association in metastatic PC, indicating context-specific coalescent behavior (Figure 1B). We examined the 6 genes in greater detail based on their ALAN profiles and again found they exhibited high concordance, but only in metastatic PC and not in benign prostate tissue (Figure 1C). Unsupervised clustering of prostate tumor biopsies (Caris, *n* = 4,490) and metastatic PC biopsies (Caris, *n* = 2,593) indicated that the expression of the 6 APUC genes was again clustered as evident by the origin at the same branch point, but interestingly not with *AR*, as it is the furthest branch point from APUC genes (Figure 1D). When we examined the expression of APUC genes in RNA-seq data from benign tissue (GTEx), at least 1 of the 6 APUC genes exhibited high expression in the adrenal gland, testis, and ovary (top 3 expressed tissues of 6 APUC genes, Figure 1E). Altogether, *HSD3B1*, *HSD3B2*, *CYP11A1*, *CYP11B1*, *CYP17A1*, and *CYP3A43*, hereby defined as APUC-6, demonstrated tissue- and cancer stage–specific interactions, with the most notable interaction in metastatic PC.

### APUC-6 genes define a subset of metastatic PCs with reduced AR activity.

**Figure 2:**
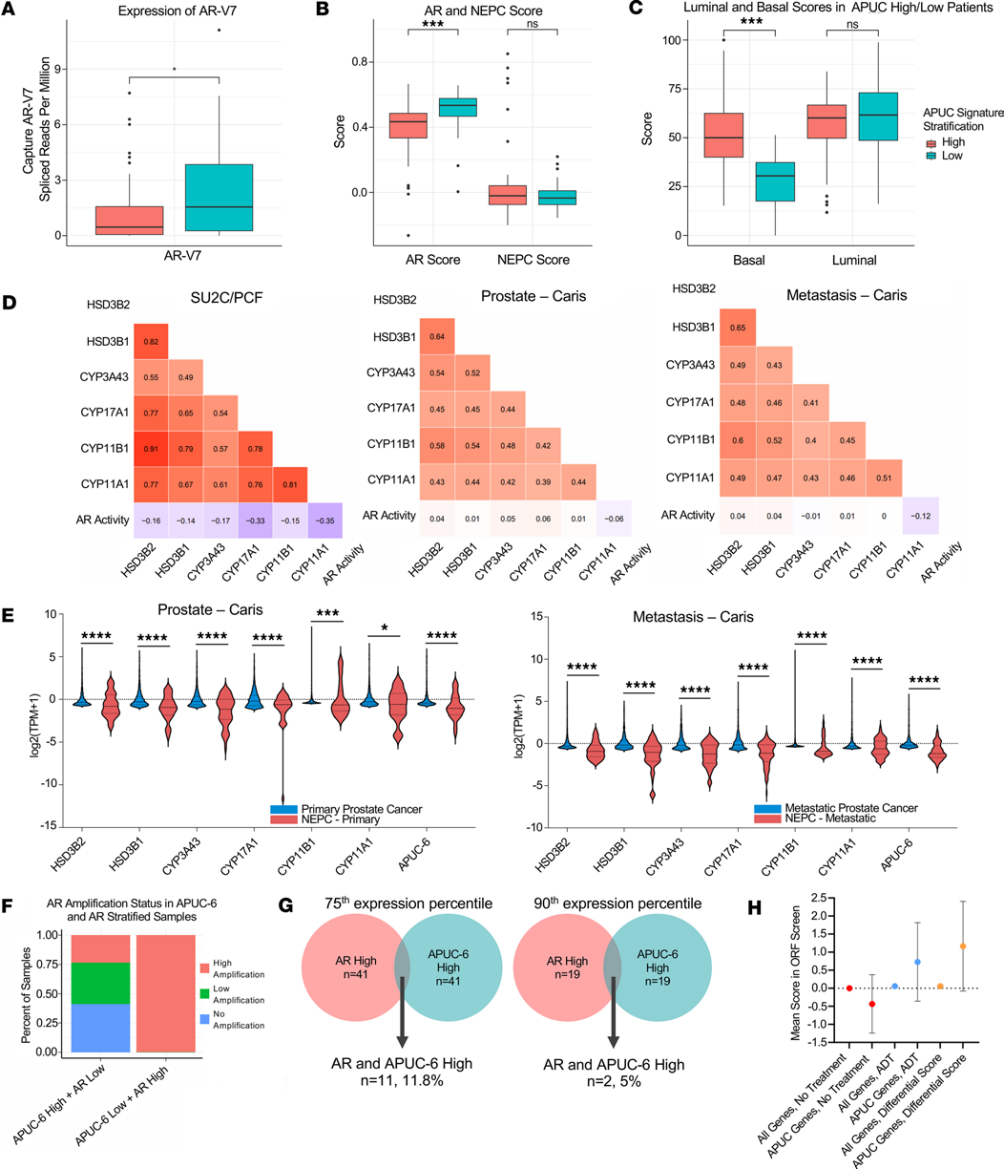
Key clinical correlates of APUC-6-high tumors. APUC-6 genes were used to stratify metastatic PC patients from the SU2C/PCF samples (27) in which we examined (**A**) the relative expression of AR-V7 (*P* value), (**B**) AR and NEPC signatures (adjusted *P* values), and (**C**) Luminal and Basal signatures (adjusted *P* values). *P* values for single tests or adjusted *P* values for multiple comparisons are shown: *P_adj_ ≤ 0.05 and *P* > 0.01, **P_adj_ < 0.01 and *P* ≥ 0.001, ***P_adj_ ≤ 0.001. NS, *P*_adj_ and *P* > 0.05. (**D**) The expression of each APUC-6 gene and AR activity was evaluated through Pearson’s correlations using the samples in Abida et al. (27) as well as the primary and metastatic samples from the Caris cohort. The correlation coefficients (*R*) are shown. (**E**) The expression of each APUC-6 gene is depicted in primary tumors that are adenocarcinomas or NEPCs. *q < 0.05, **q < 0.01, ***q < 0.001, ****q < 0.0001. (**F**) AR amplification status (no/low/high amplifications) was examined based on metastatic tumors as stratified by APUC-6 and *AR* expression. (**G**) Venn diagrams showing coexpression of *AR*-high and APUC-6-high PCs (SU2C/PCF) using 2 percentile thresholds — above the 75th and 90th percentiles of target gene(s) expression. (**H**) We aggregated the proliferation score for the 6 APUC genes based on our prior study (29). The scores are based on the *z* score of the specific gene as compared with all 17,255 genes in the overexpression screen, in which numbers reflect the standard deviation. We then presented the aggregate scores of genes based on 2 treatment conditions (No Treatment, ADT), as well as the differences in the proliferation scores for every gene (Differential Score).

As the APUC-6 genes did not exhibit the expected association with AR, we further examined the AR activity in tumors with elevated APUC-6 expression. In the SU2C/PCF study, metastatic PCs with elevated APUC-6 expression had reduced AR-V7 expression (Figure 2A) and AR activity (Figure 2B). These tumors were not associated with increased neuroendocrine PC (NEPC) scores and exhibited elevated basal- but not luminal-like profiles based on previously defined single-cell RNA-seq signatures (28) (Figure 2C). The lack of correlation with AR activity was observed even when we examined each of the APUC-6 genes in SU2C/PCF or tumors in the Caris cohorts (Figure 2D). In the Caris cohort, we found that NEPCs, as compared with the adenocarcinomas, overall had significantly lower expression of each of the APUC-6 genes (Figure 2E). When stratifying samples based on the expression of APUC-6 genes and the *AR* gene, we found that more than 75% of tumors that were APUC-6 high/*AR* low had low or no *AR* amplifications, whereas the APUC-6-low/*AR*-high group consisted of tumors that exclusively had high levels of *AR* amplifications (Figure 2F). In these samples, APUC-6 high or low status was not associated with significant differences in *AR* amplification status (Supplemental Figure 1A; supplemental material available online with this article; https://doi.org/10.1172/jci.insight.183158DS1). To establish which patients exhibited high *AR* expression or high APUC-6 expression, we determined the number of patients with high *AR* expression (>75th, >90th percentile; *n* = 52, *n* = 21) and high APUC-6 expression (>75th, >90th percentile; *n* = 52, *n* = 21) in the SUC2/PCF dataset. At the 75th percentile threshold, 11.8% (*n* = 11) of patients coexpressed these signatures, while only 5% (*n* = 2) coexpressed these signatures using a 90th percentile threshold (Figure 2G).

We next evaluated the functionality of the APUC-6 genes in cells based on a prior overexpression screen in AR-dependent lymph node carcinoma of the prostate (LNCaP) cell lines that included 17,255 genes (29). In this experiment, the *z* scores reflect relative proliferative effects as based on standard deviation for each of the 17,255 ORFs tested. Based on *z* scores, each APUC-6 gene, with the exception of *HSD3B2*, promoted proliferation when LNCaP cells were treated with enzalutamide and cultured in androgen-stripped media (Supplemental Table 1). This supports the known tumor-promoting roles of HSD3B1 (12–15) and the rationale of inhibiting CYP17A1 with abiraterone (2) and CYP11A1 with MK-5684 (ODM-208) (11). As compared with the 17,249 other genes, the aggregate viability scores of APUC-6 genes were distinct (Figure 2H), but the pro-proliferation effects were only observed when the cells were cultured in conditions that mimicked androgen deprivation therapy (ADT) (no steroid hormones and treated with enzalutamide). Upon examining APUC-6 gene expression in tumors formed from C4-2 cells, we found that intratumoral testosterone levels generally exhibited patterns similar to aggregate APUC-6 gene expression (Supplemental Figure 1, B and C). These results support the functional relevance of APUC-6 genes in the setting of prostate tumors; however, increases in APUC-6 gene expression were found in tumors with elevated tumoral androgens, but surprisingly had reduced AR-V7 and AR activity. These results support the functional relevance of APUC-6 genes in the setting of ADT treatment; however, APUC-6 genes were surprisingly associated with reduced AR-V7 and AR activity.

### APUC-6 genes exhibit robust associations with ESR1, ESR2, and PGR in prostate and metastatic biopsies.

**Figure 3:**
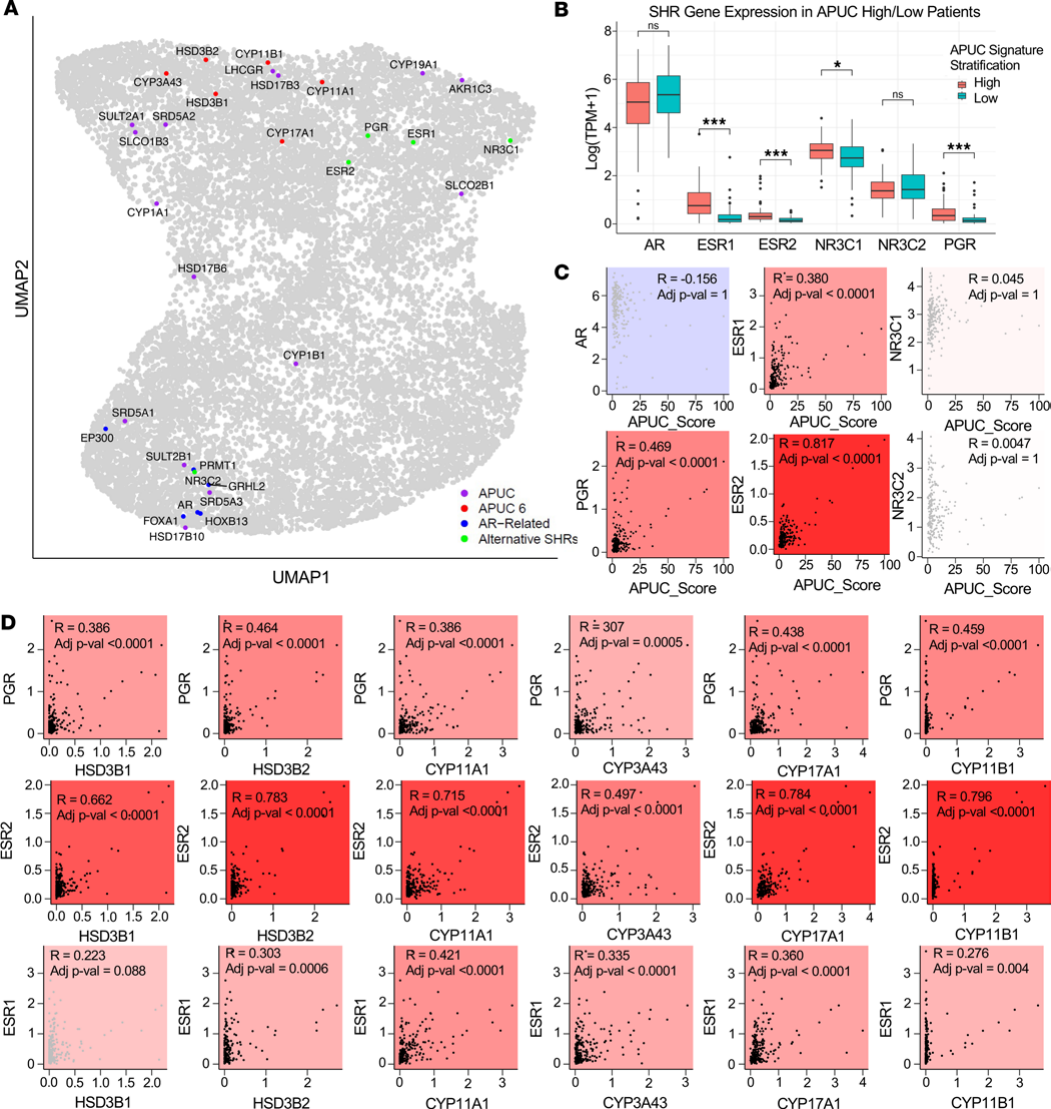
APUC-6 genes are associated with the expression of alternative hormone receptors (*ESR1*, *ESR2*, *PGR*) instead of *AR*. (**A**) UMAP was used for dimensional reduction of the ALAN outputs from metastatic PC patients, in which the distance between genes (gray dots) indicates the similarity of ALAN gene behavior. Four groups of genes are specifically labeled as APUC (purple), APUC-6 (red), AR-Related (blue), and Alternative SHRs (green). (**B**) Based on stratifying patients by APUC-6 expression, we examined the relative expression of cancer-related hormone receptors. (**C**) Pearson’s correlation was used to examine the relative expression of hormone receptors with respect to APUC-6 genes (APUC-Score) in metastatic PC samples. (**D**) *ESR1/2* and *PGR* expression levels were correlated with each APUC-6 gene. In **C** and **D**, the correlation coefficient (*R*) and adjusted *P* values (FDR adjusted for multiple comparisons) are shown: *P_adj_ ≤ 0.05 and *P* > 0.01, **P_adj_ < 0.01 and *P* ≥ 0.001, ***P_adj_ ≤ 0.001. NS, *P*_adj_ > 0.05.

In order to identify the relative interaction of APUC-6 genes with respect to all detectable genes in metastatic PC, we next conducted a dimensional reduction of ALAN outputs (Figure 3A). This approach allowed us to visualize which genes behaved similarly to the APUC-6 genes. Here we found that APUC-6 genes were indeed in close proximity on the UMAP, but were distant from *AR* and AR cofactors, including *HOXB13*, *FOXA1,* *GRHL2*, *PRMT1*, and *EP300*. Surprisingly, when examining all alternative SHRs, including *ESR1/2* (estrogen receptors, ERs), *PGR* (progesterone receptor, PR), *NR3C1* (glucocorticoid receptor, GR), *NR3C2* (mineralocorticoid receptor, MR), APUC-6 genes exhibited the most similarity to *ESR1*, *ESR2*, and *PGR*. Interestingly, *ESR1* is a current therapeutic target in breast cancers. When revisiting the metastatic tumors stratified by APUC-6 levels, we found that tumors with high APUC-6 expression had increased expression of *ESR1*, *ESR2*, and *PGR*, but reduced expression of *AR* (Figure 3B). Using APUC-6 genes as a signature, we determined that the associations with *ESR1*, *ESR2*, and *PGR* were positive and significant (adjusted *P* value < 0.0001), while the associations with *AR*, *GR*, and *MR* were not significant (Figure 3C). Furthermore, we examined whether each APUC-6 gene was associated with *ESR1/2* and *PGR*, which confirmed a positive correlation between each APUC-6 gene and *ESR1/2* and *PGR* expression (Figure 3D).

**Figure 4:**
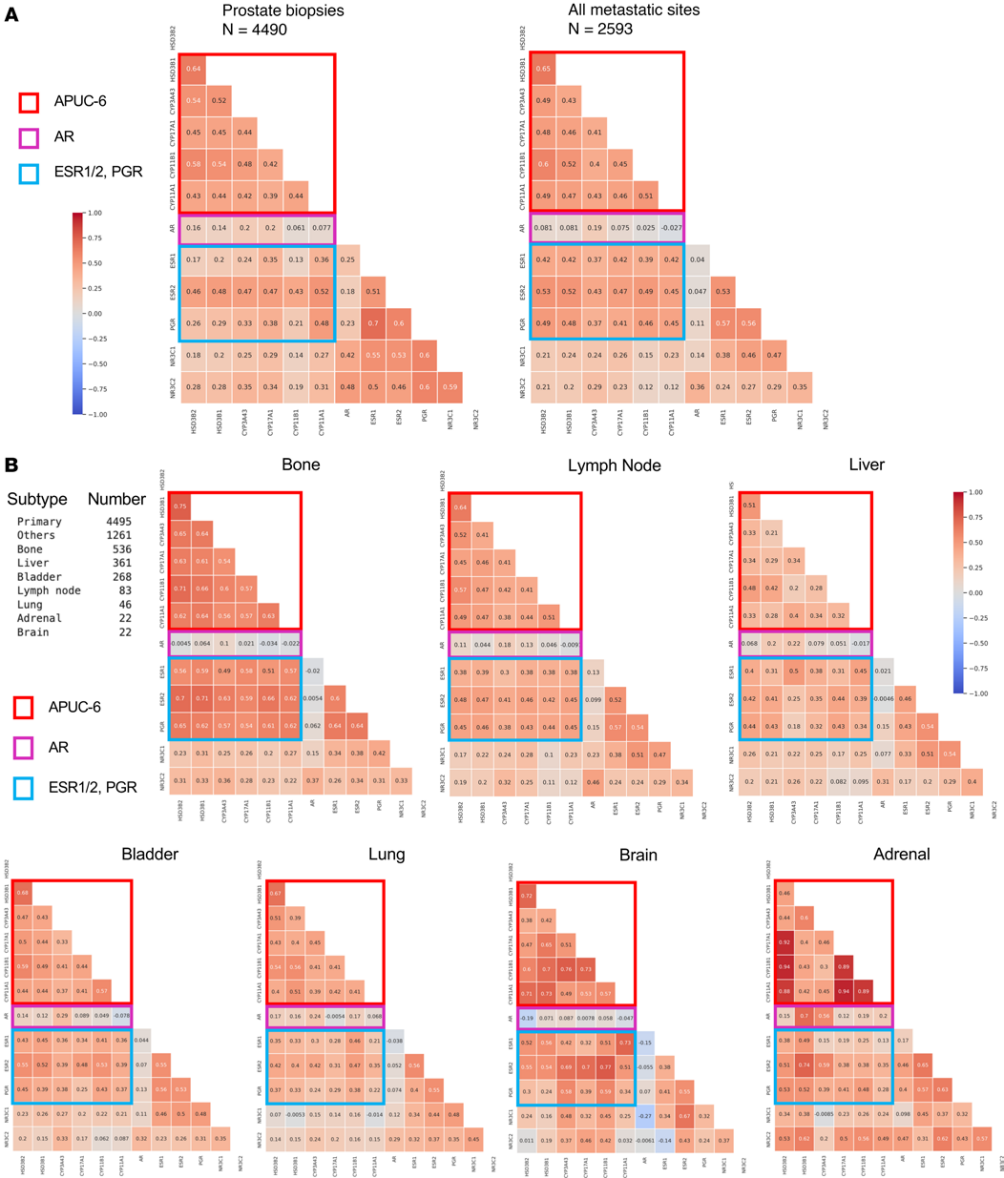
APUC-6 genes have positive associations with *ESR1*, *ESR2*, and *PGR*, but not *AR*. (**A** and **B**) In the Caris cohort of samples, we examined the Spearman’s correlation between APUC-6 genes, *AR*, ER (*ESR1*/*2*), PR (*PGR*), GR (*NR3C1*), and MR (*NR3C2*). We present the overall results as a correlation matrix and separately examined tissue from prostate tumors and metastatic PCs (**A**) and specific biopsy site (**B**). Positive correlation values are indicated in red (+1) and negative correlation values in blue (–1). Groups of genes are highlighted on each plot, including APUC-6 (red), *AR* (purple), and *ESR1/2*, *PGR* (blue).

We next aimed to confirm the relationship between APUC-6 genes with the expression of all hormone receptors in the larger Caris dataset. To do so, we cross-correlated the expression of each APUC-6 gene with the expression of all other SHRs. In the 4,490 prostate tumor biopsies, APUC-6 genes exhibited a robust correlation with one another, and all had strong positive correlations with *ESR1*, *ESR2*, and *PGR* (Spearman’s *R* 0.13–0.52), while the correlation with *AR* was relatively weak (Spearman’s *R* 0.06–0.20). In the 2,593 metastatic samples, we observed similar associations among the APUC-6 genes, but with greater correlation with *ESR1*, *ESR2*, and *PGR* (Spearman’s *R* 0.37–0.53), while the association with *AR* was further diminished (Spearman’s *R* –0.03 to 0.19) (Figure 4A). We next conducted the same analysis upon stratifying samples by metastatic biopsy sites, including lymph node (*n* = 833), bone (*n* = 533), liver (*n* = 360), bladder (*n* = 313), lungs (*n* = 114), brain (*n* = 24), and the adrenal gland (*n* = 22) (Figure 4B). APUC-6 genes exhibited robust correlation regardless of metastatic tissue sites, with the most robust association in the 22 adrenal gland samples. However, we also note that normal adrenal gland tissue also had the highest expression of APUC-6 genes (Figure 1E). The 3 SHRs *ESR1*, *ESR2*, and *PGR* exhibited the strongest correlation (Spearman’s *R* 0.49–0.71) with APUC-6 genes in the 533 bone metastasis samples. Similar to what we found in the SU2C/PCF cohort, APUC-6 genes exhibited the strongest correlation with *ESR2* across all metastatic sites. The only tissue site in which APUC-6 genes had a notable *AR* correlation was in the adrenal gland, in which we also noted that APUC-6 genes are generally expressed in the benign prostate tissue (Figure 1E). When we stratified patients based on self-reported race (European American, African American, Asian Pacific Islander), the degrees of associations showed consistent trends across populations (Supplemental Figure 2). Altogether, APUC-6 genes exhibited consistent association with each other in prostate tumor biopsies and all metastatic tumor biopsies regardless of tissue sites. They generally had a positive association with *ESR2*, *ESR1*, and *PGR* across all metastatic sites and had limited to no association with *AR*.

### Pathway analysis indicates that APUC-6 genes exhibit divergent signaling as compared with AR.

**Figure 5:**
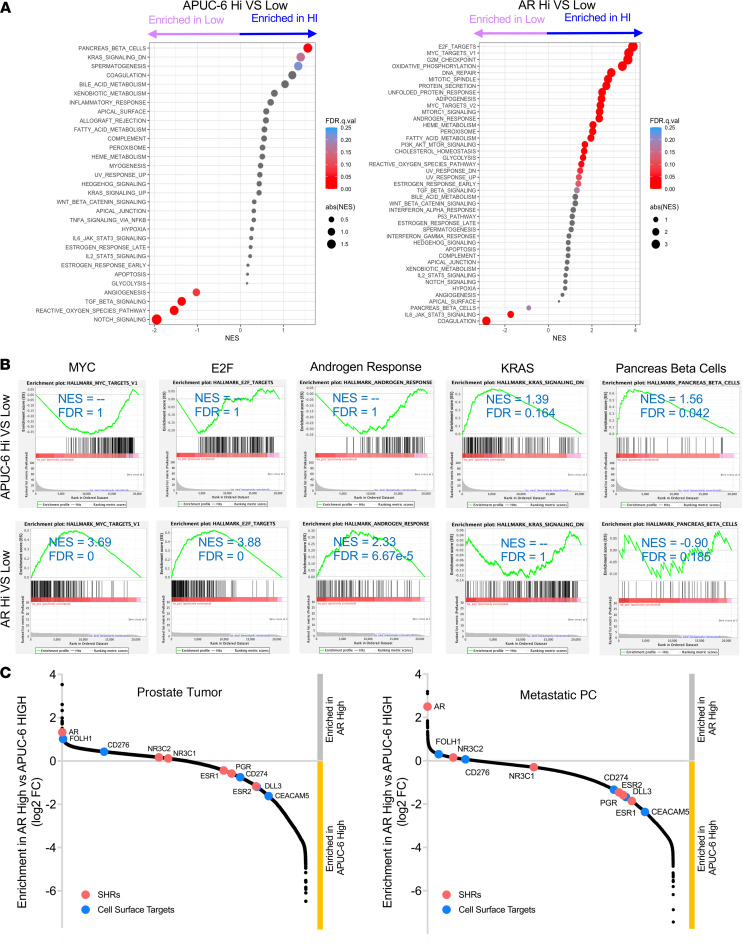
Pathway analysis indicates APUC-6 high and *AR* high regulate distinct pathways. (**A**) 50 Hallmark signatures were analyzed using GSEA based on APUC-6 high or *AR* high status. The analysis was conducted based on primary tumor samples from the Caris cohort. (**B**) The enrichment plots are shown along with net enrichment scores (NES) and FDR. (**C**) We examined the relative rank of all genes based on the differential expression upon grouping samples based on APUC-6 or *AR* expression status in primary tumor biopsies and metastatic tumor biopsies via snake plot. Genes enriched in *AR*-high tumors have a positive enrichment score (gray) and APUC-6-high tumors have a negative enrichment score (yellow). Steroid hormone receptors (red) and cell surface targets (blue) are highlighted.

To examine the biological processes that are associated with *AR*- or APUC-6-high tumors, we generated differential expression profiles and then examined the signaling pathways that are associated with these tumors using gene set enrichment analysis (GSEA) (30). In prostate biopsies (Caris), we found that APUC-6 genes were associated with increases in Hallmark signatures, including Pancreas Beta cells and KRAS signaling (Figure 5A). Here, *AR*-high tumors were expectedly associated with the Hallmark Androgen Response, MYC, and cell cycle (E2F) pathway. When we examined the GSEA enrichment plots for key pathways, including MYC, E2F, Androgen Response, KRAS, and Pancreas Beta cells, we noted that the directionality of enrichment of these pathways was exactly opposite when comparing the APUC-6-high or *AR*-high tumors (Figure 5B). Next, we further examined the magnitude in which genes were differentially expressed in APUC-6-high or *AR*-high tumors and determine whether these relationships differed between prostate biopsies and metastatic PC. In APUC-6 tumors, we indeed found reduced relative expression of B7-H3 (*CD276*) and PSMA (*FOLH1*). In the same analysis, APUC-6 genes had significant differences in the expression of *DLL3* and *CEACAM5*, both biomarkers and clinical targets implicated in advanced PCs with neuroendocrine-like features (Figure 5C) (31, 32). Furthermore, this remains true in both the prostate tumor and metastatic PC setting. As with the signaling pathways, we saw that *AR*-high tumors essentially had reciprocal relationships with the expression of each of these genes. While our interrogation of the transcriptomes does not fully explain the mechanisms that distinguish tumors with high APUC-6 and *AR*, this strongly supports the notion that these tumors have opposing gene expression patterns and should be considered a distinct subset of patients.

### APUC-6-high exhibited distinct outcomes as compared with AR-high tumors.

**Figure 6:**
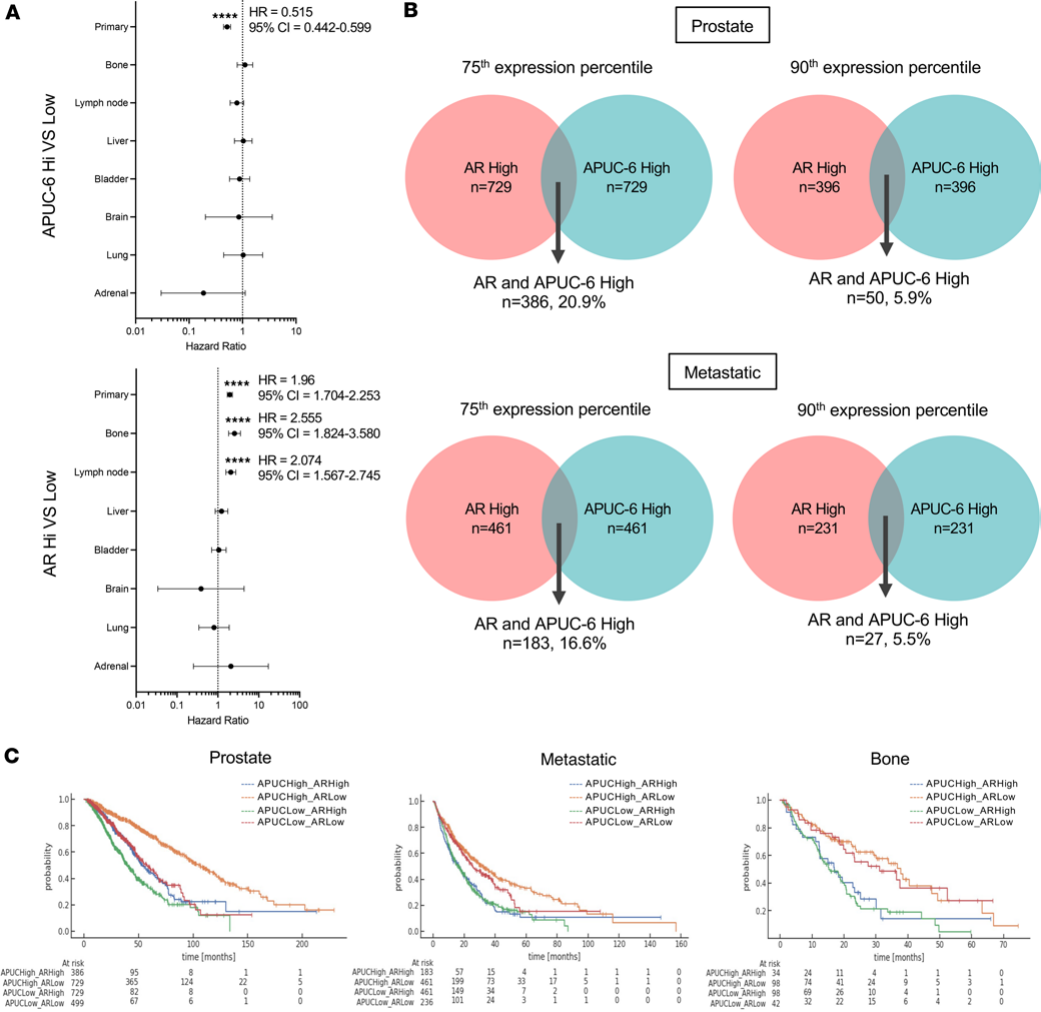
APUC-6-high and *AR*-high tumors have distinct clinical outcomes. (**A**) OS is shown for APUC-6-high or *AR*-high PC tumors based on biopsy site. ****P < 0.0001. Cox’s proportional hazards regression model and the log-rank statistic to determine significance were used to make these plots. (**B**) Venn diagrams showing coexpression of *AR*-high and APUC-6-high prostate and metastatic (top and bottom, respectively) biopsies using 2 percentile thresholds — above 75th and 90th percentiles of target gene(s) expression. (**C**) OS is shown for APUC-6 and *AR* expression status (4 combinations: APUC-6 high/*AR* high, APUC-6 high/*AR* low, APUC-6 low/*AR* high, APUC-6 low/*AR* low) across PC tumors based on biopsy site.

In prostate tumor biopsies (Caris cohort), patient tumors with high APUC-6 had improved overall survival (OS) as compared with low APUC-6 (HR = 0.515, 95% CI = 0.442–0.599, *P* value < 0.0001) (Supplemental Figure 3). In the same subset of tumors, this effect was more robust compared with tumors with high expression of *ESR1*, *ESR2*, and *PGR* (HR = 0.744, 95% CI = 0.648–0.854, *P* value < 0.0001). Interestingly, *AR*-high tumors exhibited the opposite outcomes (HR = 1.96, 95% CI = 1.704–2.253, *P* value < 0.0001) in these samples. When we examined the aggregate of all metastatic tumors or bone metastasis, APUC-6-high tumors no longer exhibited significant differences in outcomes (Figure 6A). In this setting, patients with *AR*-high tumors had worse outcomes when considering all prostate tumor biopsies (HR = 2.0, 95% CI = 1.7–2.3, *P* value < 0.0001) or bone (HR = 2.6, 95% CI = 1.8–3.6, *P* value < 0.0001) and lymph node metastasis (HR = 2.1, 95% CI = 1.6–2.7, *P* value < 0.0001). Similar to the analyses in the SU2C/PCF cohort, high expression of *AR* and APUC-6 genes was largely mutually exclusive in the Caris dataset, and only a minority of prostate or metastatic samples (5.9% and 5.5%, respectively) shared elevated expression of AR and APUC-6 genes (Figure 6B). When conducting a survival analysis in these metastatic PCs, we noted that tumors with high APUC-6 expression exhibited trends toward improved outcomes (Supplemental Figure 4). Using this knowledge, we established 4 patient groups based on *AR* and APUC-6 expression (APUC-6 high/*AR* high, APUC-6 high/*AR* low, APUC-6 low/*AR* high, APUC-6 low/*AR* low) across prostate, metastatic, and bone biopsies (Figure 6C). In all analyses, patients with the APUC-6-high/*AR*-low tumors (orange line) had the best OS, whereas patients with APUC-6-low/*AR*-high tumors (green) had the worst outcomes.

**Figure 7:**
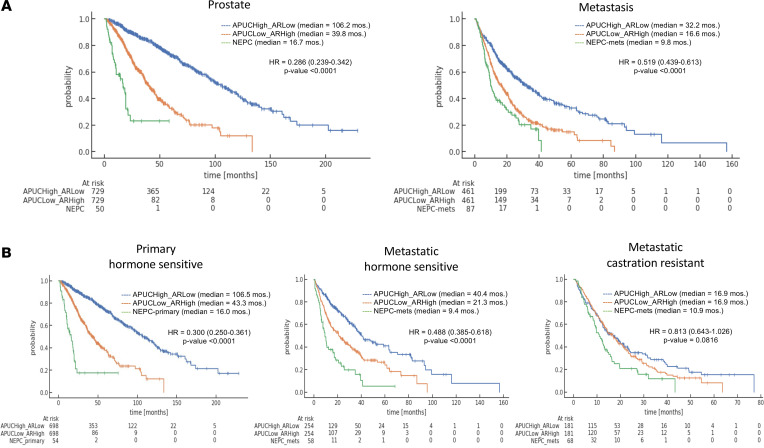
APUC-6-high, *AR*-high, and NEPC tumors have distinct clinical outcomes. (**A**) OS is shown by NEPCs and PCs organized by APUC-6 and *AR* expression status (2 combinations: APUC-6 high/*AR* low versus APUC-6 low/*AR* high). The results are illustrated based on biopsy site. (**B**) OS based on hormone sensitive and castration status as well as NEPCs or PCs after stratification by APUC-6 and *AR* expression. The samples are organized based on prostate or metastatic biopsies.

We then selected for patients with APUC-6-high/*AR*-low and APUC-6-low/*AR*-high tumors and directly compared these groups against one another and against samples pathologically annotated as NEPCs, which also have low *AR* expression. Here, patients with APUC-6-high/*AR*-low PC had significantly longer OS compared with tumors that were APUC-6 low/*AR* high or NEPC histology across biopsy sites (prostate biopsies, HR = 0.286, 95% CI = 0.239–0.342, *P* value < 0.0001; metastatic biopsies, HR = 0.519, 95% CI = 0.439–0.613, *P* value < 0.0001; bone, HR = 0.433, 95% CI = 0.294–0.640, *P* value < 0.0001) (Figure 7A). We next stratified all the samples based on hormone-sensitive and castration-resistant status, as conducted in our prior study (based on receiving less or more than 90 days of ADT) (Figure 7B) (33). In hormone-sensitive primary tumors, APUC-6 high/*AR* low exhibited robust differences in OS (HR = 0.300, 95% CI = 0.250–0.361, *P* value < 0.0001). Notably, the median OS of APUC-6-high/*AR*-low samples was 106.5 months as compared with 43.3 for the APUC-6-low/*AR*-high samples and 16.0 for NEPC. This was also the case for hormone-sensitive metastatic tumors (HR = 0.488, 95% CI = 0.385–0.618, *P* value < 0.0001). Again, the median OS of APUC-6-high/*AR*-low samples was 40.4 months as compared with 21.3 for the APUC-6-low/*AR*-high samples and 9.4 for NEPC. However, when examining castration-resistant samples, we only observed a trend toward improved outcomes (HR = 0.813, 95% CI = 0.643–1.026, *P* value = 0.0816). Altogether, we found that APUC-6, *AR* overexpression, and NEPC status can be used to define 3 distinct subsets of PCs with different outcomes.

## Discussion

In this study, we determine that a subset of 6 APUC genes acted together to define a subset of PC that is uniquely driven by APUC variation and is phenotypically distinct from other aberrations, including those of the AR and may implicate other SHRs beyond the AR. Deploying the novel ALAN computational approach captured 6 APUC genes (*HSD3B1*, *HSD3B2*, *CYP11A1*, *CYP11B1*, *CYP3A43*, and *CYP171A1*) that, when analyzed collectively, exhibited consistent and robust associations with outcome in prostate biopsies or metastatic PC. Notable among these critical 6 genes are *CYP171A1*, the target of abiraterone, *CYP11A1*, the target of opevesostat, and *HSD3B1*, which has already shown to have established impacts on patient outcomes. Altogether, these data suggest the existence of a new subset of PC that is uniquely dependent on APUC and is distinct from those with AR aberrations or AR-high activity.

Several key observations merit further discussion and evaluation. When we examined 7,083 tumor samples from the prostate biopsies and metastatic PCs analyzed by clinical grade genomic tests by Caris, these genes consistently exhibited robust coexpression. The 6 APUC genes (APUC-6) were not associated with the expression of *AR*, AR-V7, or AR activity, were enriched with a basal transcriptional profile (as compared with luminal), but did not demonstrate NEPC signatures. Across all datasets, a consistent positive association of APUC-6 genes with each other as well as *ESR1*, *ESR2*, and *PGR* was detected. Using both public and Caris datasets, we were able to stratify 2 distinct subsets of PC patients based on *AR* or APUC-6 expression, in which APUC-6-high PCs had improved outcomes. *AR* overexpression was expectedly associated with worse outcomes. Our work also supports a deeper investigation into the effects of ER and PR activity in PC patients, which has been reported to regulate circulating testosterone levels (34) or modulate key signaling pathways such as MYC in PC models (35). To this degree, the function of ER and PR in PCs may be non-canonical and instead may promote activity of other oncogenes critical for tumor progression.

Singular germline variants of APUC genes are known to regulate AR signaling. Most notably, 3βHSD1 function is enhanced by a missense-encoding variant (1245A→C), commonly detected in PC patients. PC patients with the *HSD3B1* (1245C) allele experience unfavorable rates of progression-free survival, metastasis-free survival, and OS from PC due to the enhancement of a missense-encoding variant that drives ADT resistance (14). The APUC-6 genes include *HSD3B1* and appear to exhibit coexpression in essentially all major biopsy sites. Yet, these APUC-6-high tumors instead exhibited reduced *AR* and AR-V7 expression as well as AR signatures and importantly reflected tumors with better OS. In explaining these observations, we note that the convergent behavior of APUC-6 genes was specifically observed in metastatic PCs, or PC patients with a clinical condition that merited further genomic testing. This patient population is more likely to have high-volume disease, to have already progressed on ADT, or to present with intrinsic resistance. It is thus surprising and important to discover that APUC-6 represents a patient population with better OS, with potentially unique vulnerabilities especially in the metastatic disease setting. Related to this, we also detected differences in the association of APUC-6 genes when comparing the samples from TCGA to metastatic PCs and prostate biopsies from the Caris dataset. TCGA samples are exclusively primary prostate tumors, in which fewer patients in this cohort eventually develop metastasis. Our findings indicate that APUC-6 genes, including *HSD3B1*, may have a distinct effect in patients with high-volume disease compared with localized or untreated disease. Of note, Hearn et al. have indicated that the adrenal-permissive *HSD3B1* variant was not associated with clinical outcomes in the setting of high-volume disease (15). Altogether, it is important to recognize that APUC genes, particularly the 6 resulting from our analysis, may hold the greatest impact in treatment resistance, as opposed to localized or untreated disease, a hypothesis that requires further study. It is thus a limitation of this analysis that we cannot make definitive statements implicating this network of enzymes in tumorigenesis of localized disease.

While APUC-6 genes were associated with reduced AR activity in late-stage PC, we found exceptions to this observation. As we examined tumor sites within the 22 adrenal metastasis samples, it was clear that *HSD3B1* and *CYP3A43* exhibited robust association with *AR* expression (*R* = 0.7 and 0.56), whereas the rest of the APUC-6 genes did not. This suggests that there are tissue-specific effects in the adrenal metastatic PCs that lead to distinct signaling mechanisms as compared with other metastatic sites. Outside of the APUC-6 genes, the analysis through ALAN indicated that in metastatic PC, *HSD17B10*, *SRD5A3*, and *SULT2B1*, unlike APUC-6 genes, exhibited positive associations with *AR*. This indicates that APUC genes other than the 6 we have examined may still regulate AR activity and thus could still be considered adrenal permissive. Relevant to our nomination of APUC-6, there were also other APUC genes that exhibited positive correlations with one or more of the 6 APUC genes. Particularly, *LHCGR* and *HSD17B3* had positive associations with several APUC-6 genes as well as *ESR1*, *ESR2*, and *PGR*. Upon further validation and modeling in prospective studies, expression of the APUC-6 gene set could merit incorporation as a biomarker into prognostic models such as those developed by Halabi and colleagues (36). As novel therapies are developed that target androgen production, further consideration could be given to utilization of APUC-6 as a patient selection or predictive biomarker with a goal of enhanced benefit of androgen synthesis inhibition in those patients whose tumors have upregulated this process. Altogether, future studies should continue to investigate other APUC genes and their regulation of hormone signaling.

Additional clinical implications of APUC-6-high tumors are considerable, including targeting of cell surface receptors. Work by us and others has indicated that B7-H3 expression is driven by AR signaling (37, 38). PSMA, the target of 177Lu-PSMA-617 and other approaches under investigation, is AR dependent (39). In APUC-6 tumors, we indeed found reduced relative expression of B7-H3 and PSMA. In the same analysis, APUC-6 genes had significant differences in the expression of *DLL3* (31) and *CEACAM5* (32), both biomarkers and clinical targets implicated in advanced PCs with neuroendocrine-like features. Notably, when examining the enrichment of the same genes with respect to *AR*-high tumors, the results were entirely opposite. Here, we also found that APUC-6 tumors had increased basal-like signatures, but not the neuroendocrine signature. Additionally, none of these APUC-6 tumors are associated with neuroendocrine histology. The mechanism and implications of these observations remain unclarified. Future studies may consider utilizing the APUC-6 genes to make decisions about therapeutic strategies, such as stratification in clinical studies and integration as a biomarker for selection into or away from novel therapies targeting cell surface molecules and enzymatic pathways. This provides a shorter path toward translation as compared with developing specific inhibitors against the enzymatic activity of APUC gene–encoded proteins, which regulate ligand production for SHRs.

Finally, it is notable that one of the significant APUC genes was *CYP11A1*, which encodes the most proximal adrenal enzyme that converts cholesterol to pregnenolone, catalyzing the first and rate-limiting step of all steroid biosynthesis. This finding may be of clinical relevance due to the recent development of opevesostat (formerly MK-5684, or ODM-208), an oral selective inhibitor of CYP11A1, which has shown preliminary evidence of clinical activity in patients with antiandrogen-refractory, metastatic castration-resistant PC (mCRPC) (11). In a recent phase I/II study, opevesostat produced PSA responses in a significant proportion of patients with mCRPC, but especially in those harboring activating *AR* ligand-binding domain mutations (PSA response rate, 38/64 = 59%). Interrogating the intratumoral expression of CYP11A1 (at the protein or transcript level) in these patients may also potentially refine our understanding of who may benefit most (or least) from this novel pan–steroid synthesis inhibitor.

### Study limitations.

While we examined results in both the SU2C/PCF and Caris POA cohorts, the statistical analyses included are univariate analyses and causal relationships have not been established. For examination of the Caris POA dataset, the biopsy location does not always reflect disease stage or tumor grade, as may be the case for the primary tumor biopsies. The data have been organized based on self-reported race, whereby certain patients were uncharacterized and excluded from analysis. We utilized clinical outcomes and molecular data from a heterogeneous group of patients with PC; therefore, the clinical implications of APUC-6 genes require further validation and modeling in prospective studies.

### Conclusions.

Of the many APUC genes that have been associated with the activation of AR, we found that 6 (*HSD3B1*, *HSD3B2*, *CYP11A1*, *CYP11B1*, *CYP17A1*, and *CYP3A43*) exhibit robust association in prostate and metastatic tumors and were mutually exclusive with heightened AR activity. Conversely, APUC-6 genes were instead associated with the expression of alternative hormone receptors *ESR1*, *ESR2*, and *PGR*. Prostate tumors with elevated expression of APUC-6 genes exhibited distinct outcomes as compared with those that were *AR* high and therefore represent a distinct subset of PC patients. Given that whole-transcriptome sequencing (WTS) is prevalently used for genomic testing, one should evaluate the expression of APUC-6 genes as well as *ESR1*, *ESR2*, and *PGR* in disease management of patients with PC.

## Methods

### Sex as a biological variable

This study included the analysis of genomic data from human patients. Due to the nature of PC affecting males, only male patients were included in this study. However, we do anticipate that this study is relevant to more than one sex since, as discussed throughout, genes involving female SHRs are associated with APUC-6. These SHR genes are known drivers of breast and ovarian cancer; however, the role of APUC-6 in those settings requires further investigation and was outside the scope of this work.

### Study approval

This study was conducted in accordance with the guidelines of the Declaration of Helsinki, Belmont Report, and US Common Rule. In keeping with 45 CFR 46.101(b), this study was carried out using retrospective deidentified clinical data. Therefore, patient consent was waived, and this study was considered exempt at each institutional review board.

### Specimens

We queried the Caris Life Sciences database to assess molecular changes and related survival outcomes of 7,083 prostate tumor biopsies. Comprehensive molecular profiling was performed in a CLIA/CAP/ISO15189-certified clinical laboratory (Caris Life Sciences). Samples annotated as prostate includes mixture of tumors from patients, in which some have metastatic disease but the biopsy was performed on the primary site. Metastatic samples were annotated based on biopsy location (bone, lymph node, liver, bladder, lung, and adrenal gland).

### WTS for Caris POA data

The Qiagen RNA FFPE tissue extraction kit was used for extraction. RNA quality and quantity were determined using the Agilent TapeStation (RRID: SCR_019547). Biotinylated RNA baits were hybridized to the synthesized and purified cDNA targets, and the bait-target complexes were amplified in a postcapture PCR reaction. The Illumina NovaSeq 6500 was used to sequence the whole transcriptome from patients to an average of 60 million reads. Raw data were demultiplexed by the Illumina Dragen BioIT accelerator, trimmed, counted, removed of PCR duplicates, and aligned to the human reference genome hg19 by the STAR aligner. For transcription counting, transcripts per million (TPM) molecules were generated using the Salmon expression (40).

### Public dataset preprocessing

Other than the Caris POA cohort data, all RNA-seq data were downloaded from public resources that can be found in Data availability below. Processing was done to ensure each dataset was in TPM before analyses began and each dataset required distinct pre-processing, depending on the starting format.

#### TCGA prostate.

TCGA raw read counts and associated transcript length information were downloaded using the TCGAbiolinks (version 2.30.4, project = “TCGA-PRAD”) R package (41, 42). We then subset the raw reads to include only protein-coding genes and converted to TPM, which accounted for within-sample normalization based on read depth.

#### SU2C/PCF 2019.

SU2C 2019 fragments per kilobase of transcript per million mapped reads (FPKM) data were downloaded from cBioPortal and then converted to TPM. The SU2C TPM data were further natural log-transformed for downstream analyses. The AR-V7 variant spliced reads per million expression data, AR score, and NEPC score were extracted directly from the clinical data file associated with the SU2C dataset downloaded from cBioPortal, with no further modifications.

#### GTEx prostate.

The GTEx dataset was downloaded directly from the web the portal and was already in TPM, so no further conversion was necessary. Genes with zero expression across all samples were removed from the data and duplicate gene names were made unique.

### ALAN analyses

We performed ALAN and depicted outputs, as based on our prior study (24). As the units from all public resources were distinct, we converted all input RNA-seq data into TPM (see Public dataset preprocessing above), which accounted for within-sample normalization based on read depth. The 21 APUC genes were pulled from this output and visualized in 3 separate heatmaps, which employed unsupervised hierarchical clustering to group the genes in Figure 1B. ALAN profiles for each of the APUC-6 genes were extracted and are shown in the form of violin plots in Figure 1C. Uniform manifold approximation and projection (UMAP) was applied to the SU2C cohort ALAN outputs with the default parameters in Figure 3A.

### Signature scoring and stratification

Gene sets were utilized from previously defined single-cell RNA-seq signatures for Luminal and Basal subtypes and their full gene lists can be found in the original paper by Henry et al. (28). For the SU2C study, the APUC-6, Luminal, and Basal scores were calculated by taking the sum of the log(TPM + 1) expression of their respective gene sets in each sample in the cohort (*n* = 208). These scores were then scaled from 0 to 100 across all samples. Samples with APUC-6 scores in the top 25th percentile and bottom 25th percentile were used to classify the APUC-6-high (*n* = 52) and APUC-6-low (*n* = 52) patients, respectively. The AR and NEPC scores along with AR-V7 expression were previously generated in the original study and were downloaded from cBioPortal (clinical data file). For scoring tumors from the Caris database, *z* scores for genes were determined based on expression levels across patients. The average of *z* scores for either the 6 APUC genes or *ESR1/2* and *PGR* were then utilized to group patient groups for subsequent analysis. This included stratification for APUC-6-high/*AR*-high, APUC-6-high/*AR*-low, APUC-6-low/*AR*-high, and APUC-6-low/*AR*-low patient groups, which were only generated in the Caris dataset to facilitate a more fine-grained OS analysis.

### Genome-scale ORF screen analysis

We analyzed a previously published genome-scale ORF screen that was performed in LNCaP cells from Hwang et al. (29). Specifically, we compared the experimental arms conducted in control media (FCS) with androgens and androgen stripped media (CSS) containing enzalutamide for each gene. The *z* scores of APUC-6 genes were averaged as compared to all other ORFs to reflect the relative effects of each group of genes on cell proliferation after 25 days in culture. 

### Intratumoral testosterone levels

RNA-seq and intertumoral testosterone data were collected from tumors formed from C4-2 xenografts based on our prior study (43). The aggregate APUC-6 gene expression was computed upon *z*-score scaling the 6 APUC genes based on the relative expression of the 6 genes in each of the tumors.

### GSEA

GSEA (30) was conducted on all PC samples from the Caris POA cohort to analyze 50 Hallmark signatures based on APUC-6-high or *AR*-high status. The enrichment plots were generated as output along with net enrichment scores (NES) and false discovery rate (FDR) for each signature based on 1,000 permutations. We examined the relative rank of all genes based on the differential expression upon grouping samples based on APUC-6 or *AR* expression status.

### Survival analysis

We queried the deidentified real-world evidence (RWE) outcomes dataset from the Caris Life Sciences POA registry and insurance claims data. RWE OS was defined as date of treatment initiation (day 0) to either the date of death or last contact in the insurance claims repository. As previously reported, patient death was assumed for any patient without a claim for more than 100 days, which holds true for more than 95% of patients with a recorded death in the National Death Index (NDI). Cox’s proportional HRs were calculated for each comparison group and significance was determined as *P* values of less than 0.05 using the log-rank statistic.

### Statistics

Statistical significance was determined using χ^2^ and Mann-Whitney *U* tests, with corrections for multiple comparisons where appropriate using the Benjamini-Hochberg method to control the FDR at a significance level of α equal to 0.05. For determination of statistical significance in signature scoring, a *t* test was used when data were normally distributed, whereas Wilcoxon’s test was used when data were not normally distributed, as was the case for some comparisons in Figure 2C. Differential gene expression was analyzed using the Limma R package (https://bioconductor.org/packages/release/bioc/html/limma.html) (*q* < 0.001, logFC > 1.5, –log_10_ FDR > 20). Differential gene expression was analyzed by the Mann-Whitney *U* test for prostate tumors from Caris. APUC-6 Q4 versus Q1, and *AR* Q4 versus Q1 were tested. For the box-and-whisker plots, the bounds of the boxes represent the interquartile range (IQR), or the range between the 25th and 75th percentiles, the lines within the boxes represent the median, the whiskers represent the smallest and largest values in the data within 1.5 times the IQR from the 25th and 75th percentile bounds, respectively, and any outlying values are represented as individual patient samples beyond the whiskers.

### Data availability

#### Datasets derived from public resources.

These resources are provided within the article and as follows. (a) RNA-seq data from the SU2C/PCF 2019 dataset are available at https://www.cbioportal.org/ (FPKM) and in Dataset S1 of the original manuscript (27). (b) RNA-seq data from the Prostate Adenocarcinoma TCGA dataset were accessed through TCGAbiolinks (version 2.30.4, project = “TCGA-PRAD”) R package (41, 42). (c) RNA-seq data from GTEx (Prostate) are available through the GTEx portal (https://www.gtexportal.org/home/). (26)

#### Caris datasets.

Analysis of RNA-seq data from Caris POA was provided under the project proposal for this study. The principal investigators (JH and CJR) of this study have full access to all the data and take responsibility for the integrity of the data and the accuracy of the data analysis. The Caris data are not publicly available due to data size and patient privacy, but are available upon reasonable request.

#### Code availability.

No novel computer codes were developed as part of this study. All supporting analytic code used to generate results in this work is available upon request.

#### Supporting data.

Data used to generate figures presented in the manuscript are available in the supplemental Supporting Data Values XLS file.

## Author contributions

HEB, SM, CJ, and JH conceived the project. HEB, EB, and SM contributed equally to the work and are listed in this order as co–first authors based on their oversight and involvement in the overall project. HEB contributed to the conceptualization of the analyses, the figure design, the ALAN analyses, the writing, edits, revisions, and the submission of the full manuscript. EB contributed to the design and implementation of the analyses of public data, their respective data visualizations, and the writing of the Methods section. SM contributed to the writing of the final manuscript, specifically the Introduction. N Sadeghipour contributed to the analysis and visualization of the Caris dataset. AE contributed to the analysis and visualization of the Caris dataset and the writing of the Methods section. RP, ET, and PL contributed to the writing of the manuscript. XL contributed the experimental data and analysis measuring intratumoral testosterone in xenograph models. DMG, SMD, SH, ESA, and N Sharifi contributed to the study design. CJR and JH are co–corresponding authors on this manuscript. CJR contributed to the design of the study, the project idea, and the editing of the final manuscript. JH contributed to the design and conceptualization of all analyses, the writing, the editing, and the revision of the final manuscript. All authors contributed to the editing of the final manuscript document.

## Supplementary Material

Supplemental data

ICMJE disclosure forms

Supporting data values

## Version 1 08/29/2024

In-Press Preview

## Version 2 10/22/2024

Electronic publication
