# Supplementary material for: Androgen production, uptake, and conversion (APUC) genes define prostate cancer patients with distinct clinical outcomes
Source: JCI Insight. 2024 Oct 22;9(20):e183158. doi: 10.1172/jci.insight.183158 (PMC11530133; doi:10.1172/jci.insight.183158)
Supplement: Supplemental data [file jciinsight-9-183158-s045.pdf]

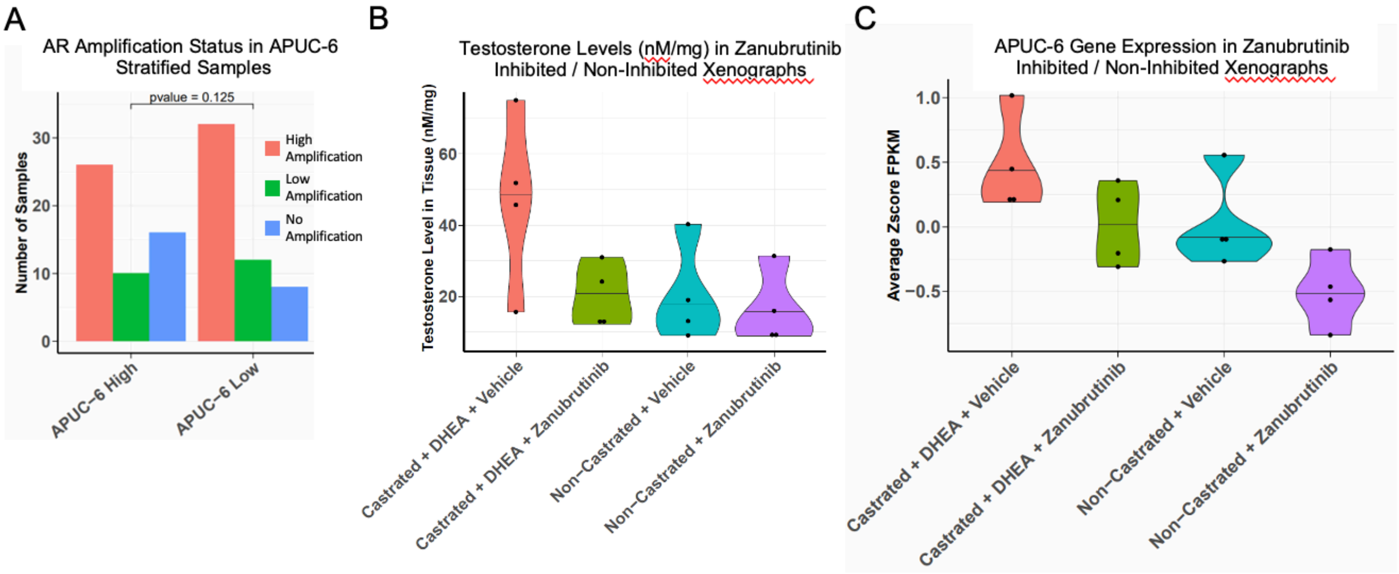

Supplemental Figure 1. (**A)** AR amplification status (no/low/high amplifications) was examined based on metastatic tumors as stratified by APUC-6 high and low status. Wilcoxon tests was applied to determine if there is a significant difference between the two groups. (**B** and **C)** The testosterone levels and RNA-seq data were collected from tumors formed from C4-2 xenografts from our prior study Li et al. 2023. The testosterone levels (**B**) and the average APUC-6 gene expression (**C**) is shown based on the 4 replicates in each experimental arm.

Race	Number
WHITE	4480
BLACK OR AFRICAN AMERICAN	1053
UNKNOWN	416
OTHER	287
ASIAN OR PACIFIC ISLANDER	189

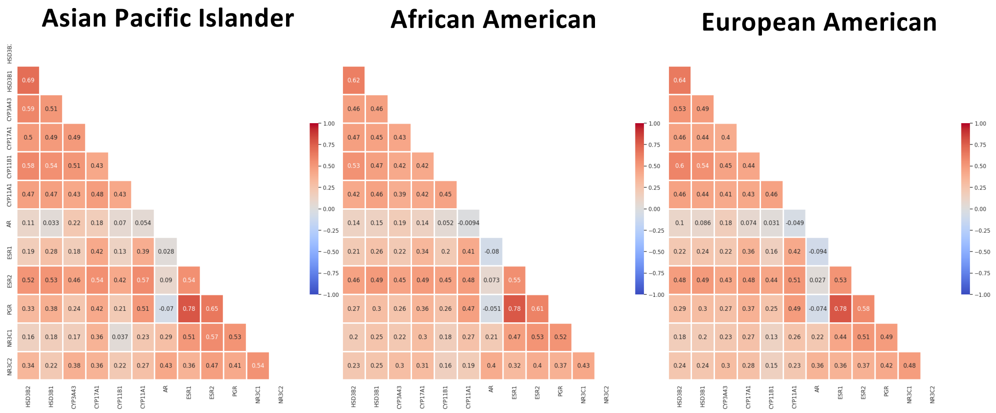

Supplemental Figure 2. Based on WTS from the Caris database, we examined the Spearman correlation between APUC-6 genes, AR, ESR1, ESR2, PGR, GR (NR3C1), and MR (NR3C2) based on biopsy sites. The number of samples are indicated based on self-reported race.

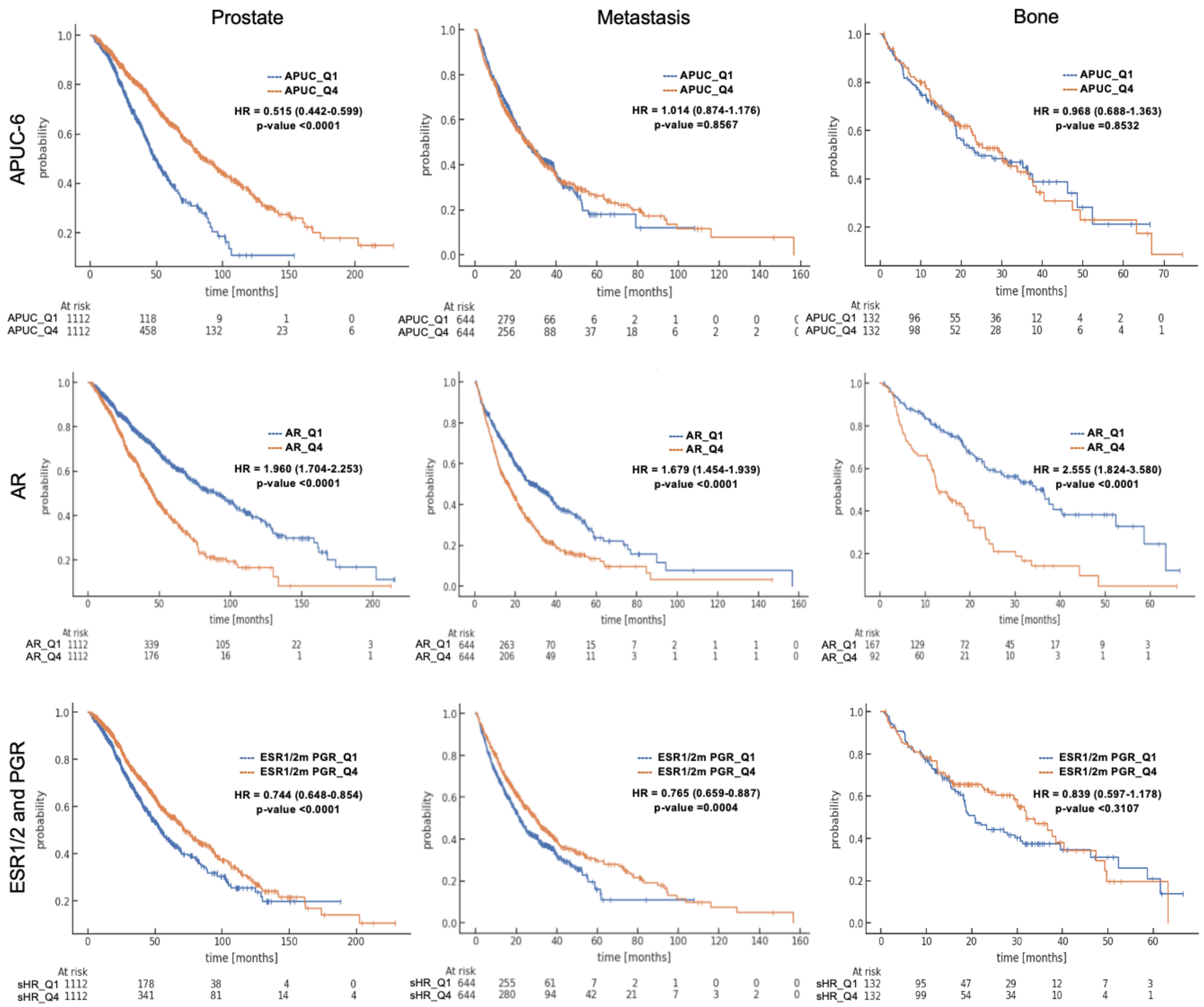

Supplemental Figure 3. Caris samples were stratified based on the aggregate expression of APUC-6, AR, or alternative hormone receptors (ESR1, ESR2, and PGR). Kaplan-Meier analysis was used to depict the effects on OS between each of the gene sets as compared to the control group.

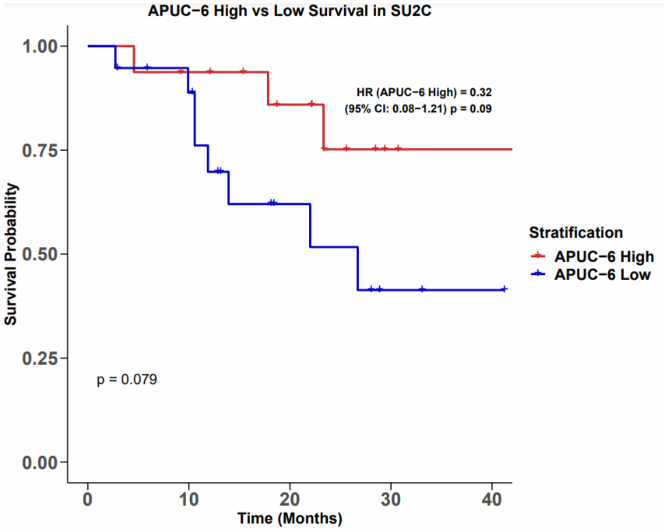

	Number of Samples	Number of Events	Median Survival Time	95% CI (Lower)	95% CI (Upper)
APUC−6 High	16	3	NA	NA	NA
APUC−6 Low	19	8	26.7	13.9	NA

Supplemental Figure 4. Of the metastatic PC samples from Abida et al. 2019, we stratified patients by APUC-6 expression high and low. The samples with available survival data were subsequently analyzed through Kaplan Meier curves.

	No treatment	ADT	Differential Score
HSD3B2	1.35	1.29	-0.06
HSD3B1	-0.94	3.03	3.96
CYP3A43	0.00	0.36	0.36
CYP17A1	-0.72	0.62	1.34
CYP11B1	-1.25	-0.34	0.90
CYP11A1	-0.77	-0.46	0.31
CYP17A1	-0.72	0.62	1.34
AVERAGE, APUC-6	-0.43	0.73	1.16
GLOBAL AVERAGE	0.00	0.06	0.06

Supplemental Table 1.
